# Influence of weight status on physical and mental health in Moroccan perimenopausal women

**DOI:** 10.11604/pamj.2016.23.153.8208

**Published:** 2016-03-31

**Authors:** Dia Eddine Oudghiri, Pilar Ruiz-Cabello, Daniel Camiletti-Moirón, María Del Mar Fernández, Pilar Aranda, Virginia Ariadna Aparicio

**Affiliations:** 1 Department of Physiology and Pathophysiology, University AbdelmalekEssaadi, Tetouan, Morocco; 2 Department of Physiology and Institute of Nutrition and Food Technology, Faculty of Pharmacy and School of Sport Sciences, University of Granada, Granada, Spain; 3 Department of Public and Occupational Health, VU University and EMGO+ Institute for Health and Care Research, Amsterdam, The Netherlands

**Keywords:** Metabolic syndrome, physical fitness, cardiovascular disease, obesity

## Abstract

**Introduction:**

There is a lack of information about fitness and other health indicators in women from countries such as Morocco. This study aims to explore the association of weight status with physical and mental health in Moroccan perimenopausal women.

**Methods:**

151 women (45-65 years) from the North of Morocco were analyzed by standardized field-based fitness tests to assess cardiorespiratory fitness, muscular strength, flexibility, agility and balance. Quality of life was assessed by means of the Short-Form-36 Health Survey. Resting heart rate, blood pressure and plasma fasting glucose, total cholesterol, LDL-cholesterol, HDL-cholesterol and triglycerides were also measured.

**Results:**

Blood pressure (P=0.001), plasma triglycerides (P=0.041) and the prevalence of metabolic syndrome (P<0.001) increased as weight status increased. Levels of cardiorespiratory fitness, upper-body flexibility (both, P<0.001), static balance (P<0.05) and dynamic balance (P<0.01) decreased as weight status increased. Pairwise comparisons showed differences mainly between normal-weight and overweight vs. obese groups. No differences between groups were observed on quality of life.

**Conclusion:**

Cardiovascular and lipid profile and fitness, important indicators of cardiovascular disease risk, worsened as weight status increased, whereas quality of life appears to be independent of weight status. Exercise and nutritional programs focus on weight management may be advisable in this under studied population.

## Introduction

A high BMI and a low physical fitness, particularly cardiorespiratory fitness, are powerful predictors of all-cause mortality, especially from cardiovascular disease (CVD) [1–3]. Moreover, loss of fitness has been shown to promote higher all-cause and CVD mortality risks regardless of BMI change [4, 5]. Regarding the association between BMI and quality of life (QoL) the findings are less clear, and studies on relationships between BMI and different measurements of mental ill-health and QoL have reported contradictory results [6–12]. Menopause is frequently associated with weight gain and a shift in body-fat accumulation from the hips and the thighs to the trunk. This android obesity can contribute to a reduction in insulin sensitivity and development of dyslipidemia, insulin resistance, and type 2 diabetes [13]. These are important risk factors for CVD, which represent the major causes of death among postmenopausal women [13]. The prevalence of overweight, obesity and metabolic syndrome (MS) among Moroccan perimenopausal women is extremely high [14, 15]. Indeed, overweight and obesity have increased considerably in the last decades among this population, especially in women, and obesity has become one of the main public health problems in the country [14, 16].

Consequently, CVD is currently the first cause of mortality among women in Morocco, as well as in Africa [17, 18]. There still being a lack of information about fitness and other health indicators in adult women from developing countries such as Morocco, especially among midlife women. Because of cultural and religion features, it could be that women in Arabic countries such as Morocco have less possibility to get involved in exercise, and its unknown to what extent this could result in higher fatness, lower fitness and therefore higher risk for developing MS and CVD. Our group has published two studies analyzing fitness in Moroccan women [15, 19]. The first one [15] described fitness and compared Moroccan with Spanish women with the same age. This was the first study performed in this topic in African women. The second study [19] was based on the relationship between cardiorespiratory fitness and the MS in this population, and the potential usefulness of fitness testing to establish MS in this specific population. However, we have not explored the influence of weight status on this population health until date. Therefore, the aim of the present study was to analyze the influence of weight status on cardiovascular and lipid profile, physical fitness and QoLin Moroccan perimenopausal women.

## Methods

A descriptive, cross-sectional design with convenience sampling was used to conduct this study. The recruitment of participants was performed by researchers from the Department of Physiology and Pathophysiology at the University Abdelmalek Essaadi, Tetouan (Morocco) via information panels, lectures, e-mails, letters or telephone. The sample was distributed between Tetouan and nearby cities like Martil. The inclusion criteria were: (a) women, (b) age ranged 45-65 years old, (c) not to have acute or terminal illness, (d) willingness to participate in the research. Most of the women belonged to two different women associations (Yad El Moussaada, and Inbiaath Niswi associations) at Tetouan and Martil (n=114), and the rest of participants sample was completed through theUniversity environment related to the present study (staff working at the Universityor their family). The final study sample comprised 151 midlife women aged 52.5±3.8 years old. A 10.6% of the women were single, 66.9% married, and 22.5% widow (any women was separated or divorced). Regarding educational level, 69.7% of the sample had no studies, 21.9% had completed the Primary school, 15.9% had finished the Secondary school, and 12.6% had a University degree. Finally, we have analyzed the socioeconomic status of the sample. The 50.3% of the sample presented a low socioeconomic level, 38.4% a medium level, and 11.3% a high socioeconomic level. Participants were informed about the study aims and procedures and signed a written informed consent to participate. All the measurements were performed by women, in a single day and by the same trained researchers to reduce inter-examiners error. The study was reviewed and approved by the Ethics Committee of the “Hospital Virgen de las Nieves” (Granada, Spain).

### Procedures

#### Anthropometry and body composition

A portable eight-polar tactile-electrode impedanciometer (In Body R20; Biospace, Gateshead, UK) was used to measure weight (kg), body fat (%) and skeletal muscle mass (kg). Height (cm) was measured using a stadiometer (Seca 22, Hamburg). Body mass index (BMI) was calculated as weight (in kilograms) divided by height (in meters) squared and categorized following the World Health Organization criteria: underweight (<18.5 kg/m^2^), normal-weight (18.5-24.99 kg/m^2^), overweight (25.0-29.99 kg/m^2^) and obese (≥30.0 kg/m^2^) [20]. Waist circumference (cm) was measured with the woman standing at the middle point between the ribs and ileac crest (Harpenden anthropometric tape, Holtain Ltd).

#### Cardiovascular profile

Systolic and diastolic blood pressure and resting heart rate were measured after 5 minutes of rest, two times, 2 minutes apart, with the person sitting down (M6 upper arm blood pressure monitor Omron. Omron Health Care Europe B.V. Hoolderdorp, The Netherlands). The average value of two trials was selected for the analysis.

#### Biochemical analysis

Fasting glucose, triglycerides, total cholesterol, low density lipoprotein (LDL)-cholesterol and high density lipoprotein (HDL)-cholesterol were measured using commercials kits (Biosystems S.A. Barcelona, Spain). Additionally, total cholesterol/HDL-cholesterol ratio was calculated.

#### Metabolic syndrome

The criteria recommended by the American Heart Association/National Heart, Lung, and Blood Institute [21] was used to establish MS. Presence of MS was considered when women met 3 or more criteria: waist circumference ≥88 cm, triglycerides ≥150 mg/dL, HDL-cholesterol <50 mg/dL, systolic blood pressure ≥130 mmHg or diastolic blood pressure ≥85mmHg, and fasting glucose ≥100 mg/dL.

#### Physical fitness

Due to the age range of the present sample, the “Functional Senior Fitness Test” battery was used because it is relatively easy to administer and requires minimal equipment, space and it is safe [22]. Additionally, we included handgrip strength and 30-s blind flamingo tests, because they are commonly used among this age group [23]. This battery assesses aerobic fitness, muscle strength, flexibility, and motor agility.

##### Aerobic fitness

The “6-min walk test” was used to assess aerobic fitness. This test measures the maximum distance (in meters) that can be walked in 6 min along a 45.7 m rectangular course [24].

##### Muscle strength

The “30-s chair stand test” measures the number of times an individual can rise to a full stand, starting from a seated position, with the back straight and feet flat on the floor without pushing off with the arms within 30 seconds. The women performed one trial after familiarization. The handgrip strength was assessed using a digital dynamometer (TKK 5101 Grip-D; Takey, Tokyo, Japan) as previously described [25]. The women performed the test twice (alternately with both hands), with 1 minute rest between measures. The best value for each hand was chosen and the average score of both hands was used for further analyses.

##### Flexibility

In the “chair sit-and-reach” test, the women started in a sitting position with one leg extended, and slowly bended forward sliding the hands down the extended leg in an attempt to touch (or pass) the toes. The number of centimeters short of reaching the toe (minus score) or reaching beyond it (plus score) was recorded. The test was performed twice for every each leg, and the average of the best value from each of them was employed in the analyses. The “back scratch test” provides a measure of the overall shoulder range of motion, and involves measuring the distance between (or overlap of) the middle fingers behind the back with a ruler. They performed the test twice, alternately with both hands, and the best value was registered. The average of both hands was used in further analyses.

##### Static balance

In the “blind flamingo” test [26], the number of trials needed to complete 30 seconds of the static position is recorded, and the chronometer is stopped whenever the person does not comply with the protocol conditions. One trial was accomplished for each leg and the average of both values was selected for the analysis.

##### Motor agility/dynamic balance

The “8-feet up-and-go” test involves standing up from a chair, walking 8 feet to and around a cone, and returning to the chair in the shortest period of time [24]. The best time from two trials was recorded and used in the analyses.

**Quality of life** The Arabic version of the Short-Form-36 Health Survey (SF36) was applied to assess QoL [27]. This questionnaire is composed of 36 items, grouped into eight scales assessing eight dimensions: physical functioning, physical role, bodily pain, vitality, social functioning, emotional role, mental health and general health. Each subscale score is standardized and ranges from 0±100, where 0 indicates the worst possible health status and 100 the best possible. In the analysis of reliability as stability of such questionnaire, correlation coefficients between the test and retest were between 0.58 for SF36-emotional role subscale to 0.99 for SF36-physical role. Internal consistency showed an alpha coefficient between alpha=0.78 for SF36-vitality subscale to alpha=0.96 for SF36-physical role subscale [28].

### Statistical analysis

The association between weight status and the study outcomes was examined by one-way analysis of covariance (ANCOVA) after adjusting for age. The model was adjusted for age due to the fact that age is strongly positively associated to worse physical fitness and lower QoL [29]. The overall P value is that reported for the main effects of the fixed factor (i.e. weight status) as provided by the ANCOVA after adjusting for age. A significant P value indicates that there are differences at least between two of the weight status groups. When significant, pairwise comparisons with Bonferroni's adjustment were performed to keep the experiment wise error rate to α=0.05 and to identify between which groups the differences were significant (e.g. normal-weight vs. obese). Chi Squared test was used to analyze differences on MS prevalence between the different weight status categories. All analyses were performed using the Statistical Package for Social Sciences (SPSS, version 16.0 for Windows; SPSS Inc, Chicago, IL), and the level of significance was set at p<0.05.

## Results

Anthropometric and body composition characteristics of the study participants are shown in Table 1. Women presented a mean BMI equal to 30.2 and an 81% of the sample was overweight or obese (any women was underweight), with an average of 96.4 cm waist circumference and a 42% body fat mass. Mean resting heart rate of the sample was 75.9 beats per minute. Mean systolic blood pressure was 132 mmHg and diastolic was 72.1 mmHg. Plasma biochemical analysis showed a mean fasting glucose concentration of 110.6 mg/dL, mean total cholesterol was 191.8 mg/dL, consisting of 48.8 mg/dL of HDL-cholesterol and 120 mg/dL of LDL-cholesterol. Mean plasma triglycerides concentration was 115 mg/dL. Fifty-seven percent of the study sample presented MS (data not shown). Cardiovascular and lipid profile of the study participants by weight status groups after adjustment for age are shown in Table 2. Systolic blood pressure was higher as weight status increased (p=0.001). Pairwise analysis showed differences between the normal-weight compared to the obese group (p=0.004) and the overweight compared to the obese group (p=0.019). Diastolic blood pressure was also higher across weight status categories (p=0.008). Pairwise analysis showed differences between the normal-weight compared to the obese group (p=0.012). Plasma fasting triglycerides increased across weight status categories (p=0.041) but without pairwise differences. The prevalence of MS also increased across weight status categories (p<0.001). No differences between groups were observed on resting heart rate and plasma fasting glucose, total cholesterol, HDL-cholesterol and LDL-cholesterol, whereas total cholesterol/HDL cholesterol index was slightly higher (borderline signification) as weight status increased (p=0.054). Physical fitness of the study participants by weight status groups after adjustment for age is shown in Table 3. Cardiorespiratory fitness was lower across weight status categories Upper-body flexibility was worse across weight status categories No differences between groups were observed on muscular fitness and lower-body flexibility. Quality of life of the study participants by weight status groups after adjustment for age is shown inTable 4. None of the SF36 subscales showed differences across weight status categories (all, >0.05).

**Table 1:** Anthropometric and body composition parameters of the study participants

Variable	Mean(standard error)	95% C.I	
		Lower limit	Upper limit
Age (years)	52.5(0.58)	51.35	53.62
Weight (kg)	75.3(1.13)	73.09	77.55
Height (cm)	157.8(0.45)	156.86	158.64
Body mass index (kg/m^2^)	30.2(0.44)	29.38	31.10
Waist circumference (cm)	96.4(1.03)	94.31	98.38
Body fat percentage (%)	42.0(0.50)	41.03	43.00
Muscle mass (kg)	23.4(0.28)	22.82	23.93
Weight status% (UW,NW,OW,OB)	0/19/33/48	-	-

Values expressed as mean(standard error), otherwise indicated; CI, confidence interval; UN, underweight; NW, normal-weight; OW, overweight; OB obese

**Table 2:** Cardiovascular and lipid profile of the study participants by weight status groups after adjustment for age

Cardiovascular and lipid profile	Normal-weight (N=29)	Overweight (N=49)	Obese (N=73)	P
Systolic blood pressure (mmHg)	124.3(3.5)^a^	128.3(2.7)^b^	138.0(2.2)^ab^	**0.001**
Diastolic blood pressure (mmHg)	68.1(1.9) a	70.7(1.4)	74.6(1.1) a	**0.008**
Resting heart rate (bpm)	74.2(1.9)	74.2(1.5)	77.6(1.2)	0.136
Glucose (mg/dL)	99.9(9.8)	106.4(7.4)	116.3(5.8)	0.288
Total cholesterol (mg/dL)	184.7(7.5)	191.7(5.5)	193.5(4.3)	0.589
HDL-cholesterol (mg/dL)	52.0(2.6)	50.1(1.9)	47.1(1.5)	0.188
LDL-cholesterol (mg/dL)	114.2(6.8)	120.7(5.1)	121.0(3.9)	0.671
Total cholesterol/HDL cholesterol index	3.7(0.3)	4.0(0.2)	4.5(0.2)	0.054
Triglycerides (mg/dL)	92.2(13.5)	104.3(10.0)	127.4(7.8)	**0.041**
Metabolic syndrome presence (%)*	14	56	72	**<0.001**

Values expressed as mean (standard error), otherwise indicated; HDL, high density lipoprotein; LDL, low density lipoprotein.

a,b Common superscripts in a same row indicate a significant difference (P<0.05) between the groups with the same letter. Pair wise comparisons were performed with Bonferroni's adjustment.

* Chi Squared test was used to analyze differences

**Table 3:** Physical fitness of the study participants by weight status groups after adjustment for age

Fitness Component		Test	Normal-weight (N=29)	Overweight (N=49)	Obese (N=73)	P
Cardiorespiratory fitness		6-minutes walking (m)	538.3(10.4)^a^	536.1(8.0)^b^	494.7(6.8)^ab^	<0.001
Muscular fitness	Upper body	Handgrip strength (kg)	25.1(0.9)	25.4(0.7)	25.1(0.6)	0.946
	Lower body	30-s chair stand (n°. stands)	14.6(0.7)	15.8(0.6)	14.2(0.5)	0.114
Flexibility	Upper body	Back scratch (cm)	-5.8(2.1)^a^	-9.9(1.7)^b^	-17.7(1.4)^ab^	<0.001
	Lower body	Chair sit-and-reach (cm)	8.3(2.1)	8.0(1.7)	5.6(1.4)	0.415
Balance	Static	30-s blind flamingo # (failures)	8.0(1.1)	8.9(0.9)	11.2(0.8)	0.041
	Dynamic/agility	8-feet up-and-go # (s)	8.0(1.1)^a^	8.9(0.9)^b^	11.2(0.8)^ab^	0.007

Values expressed as mean (standard error); # Lower scores indicate better performance.

a,b Common superscripts in a same row indicate a significant difference (P<0.05) between the groups with the same letter. Pairwise comparisons were performed with Bonferroni's adjustment

**Table 4:** Quality of life of the study participants by weight status groups after adjustment for age

Quality of life	Normal-weight (N=29)	Overweight (N=49)	Obese (N=73)	P
**SF36**				
Physical functioning	63.3(5.1)	64.0(3.8)	58.1(3.1)	0.434
Emotional Role	46.9(8.7)	44.4(6.6)	36.1(5.3)	0.453
Physical role	46.3(8.2)	35.9(6.2)	36.3(5.0)	0.542
Vitality	55.2(3.7)	52.8(2.8)	51.9(2.3)	0.762
Mental Health	45.8(3.8)	44.3(2.8)	47.4(2.3)	0.694
Social functioning	60.2(2.8)	55.7(2.1)	56.5(1.7)	0.424
Bodily pain	60.5(5.8)	52.2(4.3)	48.2(3.5)	0.194
General Health	53.1(3.7)	52.8(2.8)	51.9(2.3)	0.200

Values expressed as mean(standard error); SF36,Short-Form-36 Health Survey; # Lower scores indicate better performance; ^a,b^ Common superscripts in a same row indicate a significant difference (P<0.05) between the groups with the same letter; Pairwise comparisons were performed with Bonferroni's adjustment

## Discussion

The prevalence of metabolic syndrome, overweight and obesity among Moroccan perimenopausal women was extremely high. Blood pressure and cardiorespiratory fitness, important indicators for CVD risk, were impaired as weight status increased. Weight loss and aerobic exercise programs focus on increasing cardiorespiratory fitness and decreasing blood pressure are mandatory to reduce CVD risk in this population. To note is that QoL did not differ across weight status categories, suggesting that obesity could be not linked to QoL in this population. Prescribing exercise and diet focus on weight loss as medicine becomes an essential component. In the present study, the percentage of overweight (33%) and obese women (48%) as well as the body fat percentage (42%) were extremely elevated, but only slightly higher to epidemiologic reference values found in Moroccan women of the same age-range [14, 16, 30]. High blood pressure and lipoprotein abnormalities have been identified by many cohort studies as the major risk factors for CVD [31]. Systolic as well as diastolic blood pressure abruptly increased in our obese group. Moreover, it is well established that the MS is a powerful determinant of CVD risk, such as recurrent ischemic stroke [32] and its presence was clearly increased as weight status increased. Raised triglycerides are a component of the MS and are also strongly associated with future risk of type II diabetes as well as CVD [31]. In the present study sample, plasma triglycerides clearly increased across weight status categories. Obese women scored worse than normal-weight or overweight women in most of the functional capacity tests. Cardiorespiratory fitness is not only an objective measure of regular physical activity, but also a useful diagnostic and prognostic health indicator for patients in clinical settings [19]. Although compelling evidence has shown that cardiorespiratory fitness is a strong and independent predictor of all-cause and CVD mortality, the importance of cardiorespiratory fitness is often overlooked from a clinical perspective compared with other risk factors such as obesity [2]. Several prospective studies indicate that cardiorespiratory fitness is at least as important as the traditional risk factors, and is often more strongly associated with mortality [2]. Moreover, cardiorespiratory fitness appears to attenuate the increased risk of death associated with obesity [2].

The “6-minutes walking test” is a good marker of cardiorespiratory fitness and has been suggested as a powerful predictor of survival in some diseases [33]. The distance covered by our Moroccan women decreased as weight status increased. A recent study investigated the independent associations and the possible interaction of BMI and perceived physical fitness and functional capability with the risk of mortality in a Finnish sample. Although BMI did not prove to be an independent risk factor for mortality from CVD, perceived physical fitness and functional capability did it [3]. Moreover, active-obese people often have similar or lower risk of CVD and mortality than inactive-healthy weight people [5]. Due to the fact that maintaining or improving fitness is associated with a lower risk of all-cause and CVD mortality, preventing age-associated fitness loss could be important for longevity regardless of BMI change [4, 5]. Therefore, the present findings may suggest that recommendations regarding prevention of CVD in this population should be based on the interrelationships between physical fitness and obesity [34]. Indeed, our group has recently published, in the same sample, than those women who scored less than 480 meters in the 6-min walk test had almost 3 times higher risk for having MS [19]. To note is that the mean distance covered by the obese group was equal to 495 meters and this distance was ∼40 meters less than those walked by the normal-weight and overweight groups. Muscular strength is a predictor of functional capacity, morbidity and mortality [35, 36], however we have not confirmed a relationship between higher weight status and lower upper-body or lower-body muscular strength. Flexibility is also important in adult people and is related with lower back pain, scoliosis and is an important outcome to maintaining and restoring mobility [37]. Overweight and obese women displayed lower upper-body flexibility, which could adversely impact the functionality of these women and their daily tasks. Finally, coordinative parameters may influence daily life functioning [38]. Obesity appears to be associated with greater risk of falling in premenopausal, as well as a higher risk of greater disability in activities of daily living after a fall [39]. Overweight and obese Moroccan women displayed poor scores in static balance as well as in dynamic balance and motor agility (as assessed by the 8-feet up-and-go test). Moreover, the8-feet up-and-go test has shown to be reliable and able to differentiate patients with chronic stroke from their healthy peers [40].

Postmenopausal women are at a higher risk of hypertension, proatherogenic lipid changes, diabetes, and severe CVD as compared with their premenopausal counterparts [41]. Smoking, central obesity, blood pressure, and physical functioning are risk factors for mortality in this stage [2–4, 42]. Based on the above showed evidence, health professionals should encourage Moroccan women to participate in moderate-intensity physical activity to reduce the risk of CVD [43, 44], especially among overweight and obese women, who scored worse in most of the physical fitness outcomes studied. Based on the results of the present study, we cannot confirm a decline on QoL across weight status categories. Results found in the literature regarding weight status and mental health are inconclusive. By one hand, a raised BMI could be associated to a diversity of mental ill-health conditions such as low self-esteem, poor self-image, depression and health-adverse behaviors; however, this association appears to be higher in individuals with a BMI above 40 [8, 45]. On the other hand, in the population study by Atlantis and Baker [6], in which anxiety and depressive symptoms were the indicators of mental ill-health, the authors showed no association between BMI and mental ill-health. Huang et al.[46] investigated a large population (n=14,221) and found that BMI was associated to physical ill-health but not to mental ill-health. The lack of agreement between studies can be due to methodological issues, like the use of different questionnaires or the factors considered when the association between obesity and QoL were analyze. In a systematic review, Bacon and Aphramor [47] noticed that epidemiological studies considering the connection between BMI and mental ill-health, rarely acknowledge factors like fitness, age, gender, physical activity, nutrients intake, weight or socioeconomic status. When studies do control for these factors, increased risk of mental ill-health disappears or is significantly reduced [48, 49]. Other explanation can be that the factors affecting QoL can be different in obese people. Lund et al. [48] observed that unemployment among the morbidly obese affected their QoL more than the weight did, and Loff and Crammond [49] found that the association between BMI and mental ill-health disappeared after control for individual's overall health status. In the general population, relative increases in cardiorespiratory fitness and habitual physical activity have been associated with lower depressive symptomatology and greater emotional well-being [50]. A combined diet and exercise intervention has positive effects on QOL and psychological health, which may be greater than that from exercise or diet alone [51].

### Strengths and limitations

Some limitations need to be mentioned. i. The study lacked of a high sample size and was of convenience.ii. We have not information about the menopause's state of the women (e.g. we did not measure estradiol levels of the participants). iii. The present study was carried out only in women, and future studies should be replicated in Moroccan men. On the other hand, this is the first study examining physical fitness, fatness, cardiovascular and lipid profile and QoL across weight status in Moroccan perimenopausal women, and did it in a single report, which allowed us to draw a global picture of the physical and mental health status of the participants and the influence of obesity.

## Conclusion

Obesity is the new pandemic in Morocco. Indeed, in the present study, 81% of the perimenopausal women studied were overweight or obese (BMI > 30 kg/m2). Metabolic syndrome markers and fitness, important indicators of CVD risk, worsened as weight status increased whereas QoL appears to be independent of weight status. This study allows health professionals to understand how weight status affects health indicators such as physical fitness, MS and QoL. Since obesity and a low fitness are both associated to higher risk of CVD and all-cause of death, nutritional interventions for weight management and exercise programs focused on increasing cardiorespiratory fitness and decreasing blood pressure should be advisable in this population. Randomized intervention studies are needed to show whether nutritional and exercise interventions focus on this population induce a better cardiovascular and lipid profile. Further research is needed in African countries, in which data on health outcomes in women still scarce.

### What is known about this topic

- There is a lack of information about fitness and other health indicators in adult women from African countries, especially among women.
- The high prevalence of overweight, obesity and metabolic syndrome among Moroccan perimenopausal women is worrisome.
- Cardiovascular disease is currently the first cause of mortality among women in Morocco, as well as in Africa.

### What this study adds

- This study allows understanding how weight status affects health indicators such as physical fitness, cardiometabolic status and quality of life in perimenopausal Moroccan women.
- Metabolic syndrome markers and physical fitness, important indicators of cardiovascular disease risk, worsened as weight status increased, whereas quality of life appears to be independent of weight status in this population.
- Dietary interventions and aerobic exercise programs focus on increasing cardiorespiratory fitness and decreasing blood pressure are mandatory to reduce cardiovascular disease risk in this population.
